# Impact of hypertension severity on arterial stiffness, cerebral vasoreactivity, and cognitive performance

**DOI:** 10.1590/1980-57642016dn11-040008

**Published:** 2017

**Authors:** Henrique Cotchi Simbo Muela, Valeria A. Costa-Hong, Monica Sanches Yassuda, Michel Ferreira Machado, Ricardo de Carvalho Nogueira, Natalia C. Moraes, Claudia Maia Memória, Thiago A. Macedo, Edson Bor-Seng-Shu, Ayrton Roberto Massaro, Ricardo Nitrini, Luiz A. Bortolotto

**Affiliations:** 1 Heart Institute (Incor), University of São Paulo Medical School – Hypertension Unit São Paulo, São Paulo, SP, Brazil.; 2 Department of Physiology, Faculty of Medicine, Agostinho Neto University, Luanda, Angola.; 3 Gerontologia, Escola de Artes, Ciências e Humanidades, Universidade de São Paulo, São Paulo, SP, Brazil.; 4 Hospital Santa Marcelina, Departamento de Neurologia, São Paulo, SP, Brazil.; 5 University of São Paulo Medical School, Department of Neurology, São Paulo, SP, Brazil.

**Keywords:** hypertension, vascular changes, arterial stiffness, cerebral blood flow, cognitive performance, hipertensão, alterações vasculares, rigidez arterial, circulação cerebrovascular, desempenho cognitivo

## Abstract

Aging, hypertension (HTN), and other cardiovascular risk factors contribute to structural and functional changes of the arterial wall.

**Objective:**

To evaluate whether arterial stiffness (AS) is related to cerebral blood flow changes and its association with cognitive function in patients with hypertension.

**Methods:**

211 patients (69 normotensive and 142 hypertensive) were included. Patients with hypertension were divided into 2 stages: HTN stage-1 and HTN stage-2. The mini-mental state examination (MMSE), Montreal Cognitive Assessment (MoCA) and a battery of neuropsychological (NPE) tests were used to determine cognitive function. Pulse wave velocity was measured using the Complior^®^. Carotid properties were assessed by radiofrequency ultrasound. Central arterial pressure and augmentation index were obtained using applanation tonometry. Middle cerebral artery flow velocity was measured by transcranial Doppler ultrasonography.

**Results:**

Both arterial stiffness parameters and cerebral vasoreactivity worsened in line with HTN severity. There was a negative correlation between breath holding index (BHI) and arterial stiffness parameters. Cognitive performance worsened in line with HTN severity, with statistical difference occurring mainly between the HTN-2 and normotension groups on both the MMSE and MoCA. The same tendency was observed on the NPE tests.

**Conclusion:**

Hypertension severity was associated with higher AS, worse BHI, and lower cognitive performance.

## RESUMO.

A idade, hipertensão arterial (HA), e outros fatores de risco cardiovascular contribuem para as alterações estruturais e funcionais da parede arterial.

**Objetivo:**

Avaliar o quanto a rigidez arterial está relacionada com as alterações do fluxo sanguíneo cerebral e sua associação com a função cognitiva em pacientes com hipertensão.

**Métodos:**

Foram incluídos 211 pacientes (69 normotensos e 142 hipertensos). Os pacientes com hipertensão foram divididos em dois estágios: HA-1 e HA-2. O mini exame do estado mental (MEEM), Montreal Cognitive Assessment (MoCA) e uma bateria de testes neuropsicológicos foram usados para avaliar a função cognitiva. A velocidade da onda de pulso foi medida usando o Complior^®^. As propriedades da artéria carótida foram avaliadas usando o ultrassom de radiofrequência. A pressão arterial central e o índice de incremento foram obtidos usando a tonometria de aplanação. A velocidade de fluxo sanguíneo da arterial cerebral média foi medida pelo ultrassom com Doppler Transcraniano.

**Resultados:**

Tanto os parâmetros da rigidez arterial quanto a vasorreatividade cerebral foram piores com a gravidade da hipertensão. Houve uma correlação negativa entre o índice de apnéia e os parâmetros da rigidez arterial. O desempenho cognitivo foi pior com a gravidade de hipertensão arterial com diferença estatística ocorrendo principalmente entre os grupos HA-2 e normotensão tanto no MEEM quanto no MoCA. A mesma tendência foi observada em relação aos testes neuropsicológicos.

**Conclusão:**

A gravidade de hipertensão arterial foi associada com maior rigidez arterial, pior índice de apneia, e menor desempenho cognitivo.

## INTRODUCTION

As the world population ages, various conditions of aging, including mild cognitive impairment and dementia, are becoming increasingly prevalent and demand our immediate attention to curtail a looming epidemic.^1^ Aging, hypertension, and other cardiovascular risk factors contribute to structural and functional changes of the arterial wall.^2^^-^ 4 These changes result in decreased elasticity and increased stiffness of the arteries.^2^ Arterial stiffness, as measured by pulse wave velocity, has been associated with increased risk of cardiovascular disease including stroke.^5^^,^ 6 It has been further suggested that arterial stiffening exposes the small vessels in the brain to highly pulsatile pressure and flow and, as such, may contribute to the pathogenesis of cerebral small vessel disease.^7^ In addition, growing evidence suggests that microvascular dysfunction and damage may play an insidious, and substantial role in the overall burden of cognitive diseases.^8^ Increased aortic stiffness is associated with microvascular dysfunction, which may contribute to cognitive impairment and to overlap between vascular and cognitive risk factors.In this study, we aimed to investigate whether arterial stiffness, as measured by carotid-femoral pulse wave velocity and augmentation index, is related to the presence of cerebral blood flow changes. We also explored these associations with cognitive function in patients with different levels of hypertension compared against healthy controls.

## METHODS

In a cross-sectional study, 211 patients (69 normotensive and 142 hypertensive) were comparatively evaluated. Hypertension was defined as BP ≥ 140/90 mmHg or current use of antihypertensive drugs. Patients with hypertension were divided into 2 stages according to BP levels or medication use (HTN-1: BP 140-159/90-99 mmHg or BP under control with one or two antihypertensive drugs; HTN-2: BP ≥ 160/100 mmHg or BP under control with ≥ 3 drugs). Three groups were comparatively analyzed: Normotension, HTN stage 1 and HTN stage 2. Controlled HTN was defined as BP levels < 140/90 mmHg with the use of antihypertensive drugs. Patients from the Hypertension Unit of the Heart Institute of the University of São Paulo Medical School were consecutively recruited from June 2013 to December 2015. The normotension group participants were recruited among patients without CV disease followed yearly at the Heart Institute as part of a protocol for cardiovascular assessment.^9^

Patients that met the following criteria were excluded: age < 18 years, overt cerebrovascular disease (previous stroke or transient ischemic attack), diabetes mellitus, smoking, arrhythmias, heart failure associated with left ventricular dysfunction, known neurodegenerative or psychiatric disease, and being illiterate. Educational level was assessed by the number of completed school years. The local ethics committee approved the protocol and all participants gave written, informed consent.

Blood pressure measurement. Brachial systolic and diastolic blood pressure was measured using an Omron automatic device, HEM-705 CP model, on the right upper arm, with the subject seated, after 5 minutes of resting, following the recommendations of the VI Brazilian Guidelines on Hypertension.^10^ To determine the systolic and diastolic blood pressure (SBP and DBP) of each patient, the mean of 3 measurements taken at 1-minute intervals was calculated.

Arterial stiffness. Carotid-femoral pulse wave velocity (cfPWV) was analyzed with a noninvasive automatic device, Complior (Colson, Garges les Gonesses, France), and carotid measurements (intima media thickness [IMT] and carotid diameter) were taken with a high-definition echo-tracking device (Wall Track System, Medical Systems Arnhem, Oosterbeck, The Netherlands) by an experienced observer blinded to the clinical condition of each participant. All measurements were taken between 1:00 and 4:00 p.m., with the patient in a recumbent position while awake. The PWV measurement technique has been described previously elsewhere.^11^^-^ 13 Briefly, common carotid artery and femoral artery pressure waveforms were recorded noninvasively using a TY-306 Fukuda pressure-sensitive transducer (Fukuda, Tokyo, Japan). The pressure waveforms were digitized at the sample acquisition frequency of 500 Hz. The 2 pressure waveforms were then stored in a memory buffer. A preprocessing system automatically analyzed the gain in each waveform and adjusted it for equality of the 2 signals. When the operator observed a pulse waveform of sufficient quality on the computer screen, digitization was suspended and calculation of the time delay between the 2 pressure upstrokes was initiated. Measurements were repeated over 10 different cardiac cycles, and the mean was used for the final analysis. The distance traveled by the pulse wave was measured over the body surface as the distance between the 2 recording sites (D), whereas pulse transit time (t) was automatically determined by the Complior device; PWV was automatically calculated as PWV=D/t, and 80% of this distance defined the pulse wave traveled distance (common carotid artery-common femoral artery × 0.8).^14^ Carotid diameter and IMT were evaluated using a high-resolution echo-tracking system (Wall Track System, Medical Systems Arnhem, Oosterbeck, The Netherlands) coupled with a conventional 2-dimensional vascular echograph (Sigma 44 Kontrom Instruments, Watford, UK) equipped with a 7.5-MHz probe. Measurements were performed on the left common carotid artery 1 cm below the bifurcation at the site of the distal wall. IMT was measured at the thickest point, not including plaques, on the near and far walls with a specially designed computer program. A high IMT reproducibility rate has been previously demonstrated.^15^ Plaque was defined as a localized thickening greater than 1.2 mm that did not uniformly involve the whole artery. Aortic distension, defined as the systolic-diastolic variation, was automatically calculated. The SphygmoCor system (AtCor Medical, Sydney, N.S.W., Australia) was used to estimate central blood pressure (central systolic blood pressure [cSBP] and central diastolic blood pressure [cDBP]), Augmentation index (Alx), and Augmentation index corrected for a heart rate of 75 beats per minute (AIx75), another arterial stiffness marker. The SphygmoCor measurement technique has been described elsewhere.^16^ Briefly, applanation tonometry was performed on the left radial artery with the patient seated. This was done by lightly applying a micromanometer-tipped probe to the left radial artery over the extended wrist, compressing the vessel wall sufficiently so that transmural forces within the vessel wall were perpendicular to the arterial surface. All readings recorded were found to meet the manufacturer's quality control standards integrated into the software package.

Transcranial doppler. Cerebral blood flow velocity measurements were performed with subjects in a recumbent position while awake with the head in a neutral position. BP was continuously recorded, noninvasively, through finger plethysmography (Finometer, Finapress Medical Systems BV, The Netherlands) in the left arm positioned at heart level. Blood flow velocities were measured continuously in the middle cerebral artery (MCA) using a transcranial Doppler device equipped with a 2-MHz transducer (Doppler-Box, DWL, Germany), positioned in the temporal region.

Blood pressure and cerebral blood flow (CBF) velocity data were continuously transferred to a computer for analysis. These data were obtained at a sampling rate of 200 Hz. All signals were visually inspected to identify artifacts or noise. The spicules were eliminated by the linear interpolation method, and a frequency of 20 Hz was used as the cut-off for filtering signals. The beginning and end of each cardiac cycle were identified, and the mean values of CBF velocities and BP were calculated, beat-to-beat.

The data acquisition protocol followed a pattern to assess both static and dynamic cerebral autoregulation such that cerebrovascular parameters were taken at the beginning and the end of the following periods: rest (3 minutes), breath-holding test (30 seconds), and handgrip maneuver (3 minutes).

### Breath-holding test

The breath-holding test was performed according to Harrison and Markus' descriptions.^17^ In brief, after normal breathing in ambient air for about 4 minutes, patients were instructed to hold their breath after a normal inspiration. During the maneuver, MCA blood velocity was continuously recorded. The highest mean velocity that displayed at the end of the apnea period was considered the maximum increase in mean cerebral blood velocity. The apnea time was also recorded.

Breath-holding index (BHI): BHI was calculated as the percentage increase in the MCA mean blood velocity registered during apnea, divided by apnea time in seconds ([(Vfa – Vra)/Vra] × 100 x s^-1^), where Vfa is the MCA mean blood velocity at the end of the apnea period, and Vra is the resting (baseline) MCA mean blood velocity, and s^-1^ denotes per second of apnea.

Handgrip maneuver: This was performed with a dynamometer in the right hand of the subject. To calculate the force applied during the maneuver, the participants were instructed to squeeze the dynamometer 3 times, with an interval of 10 seconds between maneuvers, using the maximum force of muscle contraction. The values were used to calculate the mean maximum contraction force. In the experiment, the patient squeezed the dynamometer for 3 minutes, exerting an effort corresponding to 30% of the mean maximum contraction force, in line with previous publications.^18^

### Cognitive Function Evaluation

#### Mini-Mental State Examination (MMSE).

The MMSE is a commonly used 30-point scale for assessing cognitive function based on orientation, registration, attention and calculation, recall, language, and praxis. MMSE administration was performed according to existing standards.^19^ Because participants had heterogeneous educational levels, scores were adjusted for level of education. The following cutoff scores were used to identify abnormal cognition in this study: ≤ 21 for patients with < 8 years of education, ≤ 23 for those with 9–11 years of education, and ≤ 25 for those with ≥ 12 years of education.^20^

#### Montreal Cognitive Assessment (MoCA).

The MoCA is a rapid screening instrument to identify mild cognitive impairment (MCI).^21^ It assesses attention and concentration, executive functions, memory, language, visuoconstructional skills, conceptual thinking, calculations, and orientation. The total possible score is 30 points, and a score of ≥ 26 is considered normal. A previous validation study in Brazil suggested 25 points as the ideal cutoff for MCI identification.^22^ To counterbalance the effect of lower education, 1 point was added to the final score of those individuals with < 12 years of education.

#### Neuropsychological Evaluation (NPE).

Procedures and descriptions of the neuropsychological tests used in this study have been published elsewhere.^23^^-^ 31 The neuropsychological test battery included the Boston Naming Test (BNT),^23^ Rey Auditory Verbal Learning Test (RAVLT5 – sum of 5 recall trials of 15 words, RAVLT6 – immediate recall after inference, and RAVLT7 – delayed recall after 30 minutes),^27^ the Rey-Osterrieth Complex Figure Test copy and delayed recall (REY-C and REY-30),^28^ Semantic Verbal Fluency animal category (VF),^24^ Phonological Verbal Fluency (FAS),^26^ Forward and Backward Digit Span Test (FDST and BDST),^31^ Trail Making Test part A and B (TMT-A and TMT-B),^25^ Clock Drawing Test (CDT),^29^ and Digit Symbols Substitution Test (DSST).^30^

We computed scores for global cognition (mean z-score of the BNT, RAVLT5, RAVLT6, RAVLT7, REY-C, REY-30, VF, FAS, FDST, BDST, TMT-A, TMT-B, CDT, and the DSST), language (BNT), memory (mean z-score of RAVLT5, RAVLT6, RAVLT7, and REY-30), executive functioning (mean z-score of VF, FAS, BDST, and TMT-B), visuospatial abilities (mean z-score of REY-C and CDT), attention (mean z-score of FDST and TMT-A), and processing speed (DSST). Z scores were calculated using normotension as the reference group.

Statistical analysis. Data were analyzed with SPSS for Windows 21.0. The distribution of the data was determined using the Kolmogorov-Smirnov test. Continuous variables are expressed as mean and SD or as median and range if not normally distributed and were analyzed by the independent-samples *t*-test and Mann–Whitney U test, when applicable. Categorical data are expressed as percentages. Analysis of variance (ANOVA) was used with Bonferroni post hoc comparisons among the study groups. The Kruskal–Wallis test was used for categorical variables. Pearson's coefficient was used for bivariate correlations between BHI and arterial stiffness properties and multivariate step-wise analysis was performed. Statistical significance was set at 5%.

## RESULTS

This study aimed to evaluate AS and BHI in a healthy population compared against patients with hypertension of different levels of severity. Baseline characteristics are presented in Table 1. The groups were comparable regarding age, but the control group had lower BMI, SBP, DBP, and higher monthly income. The HTN-2 group had hypertension for longer (disease duration), used relatively more antihypertensive drugs and had a lower proportion of patients with BP under control. Diuretics, calcium channel blockers, and angiotensin system blockers were the most prescribed drugs.

**Table 1:** Demographic, social, and clinical characteristics of the study participants.

Variable		Normotension (69)	HTN stage 1 (83)	HTN stage 2 (59)	*p*
Age (Years), mean ± SD		52.2 ± 13.9	52.4 ± 13.2	50.9 ± 10.3	0.78
Sex, female (no, %)		38 (55.1)	47 (56.6)	31 (52.5)	0.89
Race, white (no, %)		44 (63.8)	64 (77.1)	36 (61.0)	0.07
Married, (no, %)		35 (50.7)	55 (66.3)	33 (55.9)	0.09
Weight (kg), mean ± SD		74.3 ± 16.2	77.1 ± 14.2	83.1 ± 13.7	0.004^a^
Height, (m), mean ± SD		1.7 ± 0.1	1.6 ± 0.9	1.7 ± 0.1	0.52
BMI (kg/m^2^), mean ± SD		26.7 ± 4.2	28.4 ± 4.6	30.2 ± 4.5	< 0.001^a^
Education (years), mean ± SD		13.0 ± 3.9	11.4 ± 4.4	10.0 ± 4.2	< 0.001^a^
Monthly income, median (range) (R$)		3000 (730-15000)	2000 (600-8500)	1900 (500-20000)	0.02a
SBP (mmHg), mean ± SD		121.9 ± 8.3	134.1 ± 13.9	148.1 ± 24.7	< 0.001^b^
DBP (mmHg), mean ± SD		76.5 ± 6.7	82.1 ± 9.8	91.3 ± 13.5	< 0.001^b^
HTN time (years), median (range)		–	8.0 (1-37)	10.0 (1-36)	< 0.001
Number of drugs, mean ± SD		–	1.42 ± 0.8	3.8 ± 1.2	< 0.001
BP control, (no, %)		–	47 (56.6)	21 (35.6)	0.01
Most used drugs	ARB, (no, %)	–	37 (32.5)	30 (50.1)	0.03
	ACEI, (no, %)	–	30 (36.1)	26 (44.1)	0.34
	Diuretics, (no, %)	–	38 (45.8)	52 (88.1)	< 0.001
	CCB, (no, %)	–	10 (12.0)	46 (78.0)	< 0.001
	BB, (no, %)	–	10 (12.0)	37 (62.7)	< 0.001

P value refers to comparisons of the means or proportions among the groups by the 1-way ANOVA, Kruskal–Wallis, and Mann-Whitney tests. ACEI, angiotensin-converting enzyme inhibitors; ARB, angiotensin receptor blockers; BB, beta blockers; BMI, body mass index; CCB, calcium channel blockers, DBP, diastolic blood pressure; HTN, hypertension; SBP, systolic blood pressure; SD, standard deviation; R$ (1 US dollar = 3.3 Real).

a Normotension vs. HTN stage-2.

b All groups different.

Cerebral blood flow velocities and breath-holding index are presented in Table 2. There were no differences in blood flow velocities evaluated at rest, during the apnea test or the handgrip maneuver among the groups. However, response to apnea stimulation, as represented by the breath-holding index, worsened according to severity of hypertension. In this scenario, the HTN stage 2 group had a worse breath-holding index than the control and HTN stage 1 groups.

**Table 2:** Doppler velocities and breath-holding index according to HTN severity.

Variable – mean ± SD		Normotension (57)	HTN stage 1 (72)	HTN stage 2 (51)	*p*
Resting (Middle cerebral artery)	Initial velocity (m/s)	57.2 ± 10.8	52.2 ± 13.9	56.5 ± 13.7	0.76
	Final velocity (m/s)	59.3 ± 12.8	60.3 ± 15.5	57.9 ± 13.7	0.66
Apnea maneuver	Initial velocity (m/s)	57.9 ± 12.3	59.9 ± 12.3	58.4 ± 14.4	0.68
	Final velocity (m/s)	81.5 ± 20.1	79.2 ± 18.4	75.4 ± 20.9	0.29
	Breath-holding index	1.4 ± 0.6	1.1 ± 0.6	1.0 ± 0.6	0.004a
Handgrip maneuver	Initial velocity (m/s)	59.1 ± 12.3	60.3 ± 14.3	56.9 ± 15.3	0.43
	Final velocity (m/s)	63.6 ± 15.3	63.9 ± 17.3	60.7 ± 19.0	0.57

P value refers to comparisons of the means among the groups by the 1-way ANOVA step-wise test with Bonferroni post-hoc analysis. HTN, hypertension; SD, standard deviation. asignificance difference (Normotension vs. HTN stage 1 and HTN stage 2).

Arterial properties according to blood pressure level are presented in Table 3. Hypertensive groups had worse arterial properties with differences observed especially between the control and HTN-2 groups.

**Table 3:** Arterial proprieties according to blood pressure level.

Variable – mean ± SD	Normotension (69)	HTN stage 1 (83)	HTN stage 2 (59)	*p*
Diameter, mm	6.5 ± 0.6	7.0 ± 0.8	7.2 ± 0.7	< 0.001^a^
Distension, %	4.8 ± 2.1	4.7 ± 2.2	5.0 ± 1.7	0.572
IMT, mm	0.7 ± 0.1	0.8 ± 0.1	0.8 ± 0.1	0.003^a^
PWV, m/s	7.5 ± 1.4	7.9 ± 1.2	8.0 ± 1.3	0.100
AIx	22.3 ± 12.9	24.7 ± 12.2	30.4 ± 11.9	0.001^b^
Alx75	21.1 ± 12.0	24.2 ± 9.9	25.8 ± 9.8	0.038^c^
cSBP, mmHg	111.5 ± 9.5	121.1 ± 14.6	141.1 ± 32.6	< 0.001^d^
cDBP, mmHg	76.7 ± 7.8	83.0 ± 10.5	92.7 ± 17.1	< 0.001^d^

P value refers to comparisons of the means among the groups by the 1-way ANOVA step-wise test with Bonferroni post-hoc analysis. Alx, augmentation index; Alx75, augmentation index corrected to 75bpm; IMT, intima media thickness; cDBP, central blood pressure; cSBP, central systolic blood pressure; PWV, pulse wave velocity; HTN, hypertension; SD, standard deviation.

a Normotension vs. HTN stage 2;

b Normotension vs. HTN stage 1 and HTN stage 2;

c HTN stage 2 vs. normotension and HTN stage 1;

d All groups different.

On bivariate analysis, there was a negative correlation between BHI and arterial stiffness properties, respectively: PWV (r = –131, p = 0.083), Alx (r = –193, p = 0.012), Alx75 (r = –201, p = 0.009), cSBP (r = –179, p = 0.019), and cDBP (r = –158, p = 0.039). On multivariate analysis, only Alx75 independently and negatively predicted breath-holding index, showing that for every 1 unit increase in Alx there was a 0.012 decrease in BHI (B = –0.012, 95%CI: –0.020 to –0.003, p = 0.009).

Table 4 presents the cognitive performance of groups, according to blood pressure levels. Cognitive performance worsened in accordance with severity of hypertension with a statistical difference occurring mainly between the HTN-2 and normotension groups on both the MMSE (control: 28.0 ± 2.0; HTN-1: 27.4 ± 2.1 and HTN-2: 26.9 ± 2.1, p = 0.004) and MoCA (control: 25.6 ± 3.0; HTN-1: 24.9 ± 3.0 and HTN-2: 23.8 ± 3.3, p = 0.003).The same tendency was observed on the NPE tests.

**Table 4:** Cognitive performance according to blood pressure level.

Cognitive Domain – mean ± SD	Normotension (69)	HTN stage 1 (83)	HTN stage 2 (59)	*p*
MMSE	28.0 ± 2.0	27.4 ± 2.1	26.9 ± 2.0	0.004^a^
MoCA	25.6 ± 3.0	24.87 ± 3.0	23.8 ± 3.4	0.003^a^
Global cognitive function	0.02 ± 0.6	–0.3 ± 0.7	–0.4 ± 0.7	< 0.001^b^
Language	0.01 ± 1.0	–0.3 ± 1.1	–0.5 ± 1.4	0.038^a^
Memory	0.03 ± 0.8	–0.2 ± 1.1	–0.4 ± 1.0	0.019^a^
Executive function	0.01 ± 0.8	–0.4 ± 0.8	–0.5 ± 0.6	< 0.001^b^
Visuospatial abilities	0.02 ± 0.9	–0.2 ± 0.8	–0.4 ± 1.0	0.047^a^
Attention	0.03 ± 0.7	–0.3 ± 0.8	–0.4 ± 0.9	0.007^a^
Processing Speed	0.03 ± 1.0	–0.4 ± 0.9	–0.6 ± 0.9	< 0.001^b^

P value refers to comparisons of the means among the groups by the 1-way ANOVA step-wise test with Bonferroni post-hoc analysis. HTN, hypertension; MMSE, mini mental state examination; MoCA, Montreal cognitive assessment; SD, standard deviation.

a Normotension vs. HTN stage 2.

b Normotension vs. HTN stage 1 and HTN stage 2.

On bivariate correlation between cognitive and cerebral parameters, BHI was positively associated with better performance on cognitive tests (MMSE: r = 0.156, p = 0.039; MoCA:r = 0.189, p = 0.012 and NPE: r = 0.205, p = 0.005), suggesting that patients with lower blood pressure had better cerebral vasoreactivity (CVR) and performed better on cognitive function evaluation.

## DISCUSSION

The main finding of this study was that the patients with severe hypertension had higher arterial stiffness, lower breath-holding index, and lower cognitive performance. This finding suggests that hypertension severity is associated with higher arterial stiffness that may lead to impaired cerebral vasoreactivity and lower cognitive performance. The arterial system gradually stiffens, because of the combined effects of ageing, high blood pressure, and other vascular risk factors.^3^ Several studies have been performed in the general population investigating the association between arterial stiffness and markers of cerebral small vessel disease.^30^ Our findings that arterial stiffness was associated with both decrease in cerebral vasoreactivity and lower cognitive performance are in line with these studies.

The mechanisms linking large-artery stiffness and manifestations of (silent) cerebral small-vessel disease are complex and not well understood.Moreover, contrary to large-artery stiffness, the role of small-artery stiffness has been studied less extensively.^31^ Our findings are consistent with the hypothesis that cerebral small-vessel disease can result from abnormal flow pulsations into the brain microcirculation as a consequence of aortic stiffening, linking systemic large-artery to cerebral small-vessel disease.^32^ The brain is, under normal conditions, continuously perfused at high-volume flow throughout systole and diastole.Exposure to highly pulsatile pressure and augmented flow, which exist in the carotid and vertebral arteries as a result of arterial stiffening, may, thus, lead to microvascular damage, and possibly, cognitive impairment and stroke.^32^^,^ 33

Structural and functional changes of the arterial wall, caused by aging and cardiovascular risk factors, result in decreased elasticity and increased stiffness of the arteries.^3^^,^ 4 Vascular resistance and pulse wave reflections are very low in the brain and kidney microcirculation.^32^ Therefore, arterial stiffening exposes the brain and kidney small vessels to highly pulsatile pressure and flow. It is hypothesized that these abnormal flow pulsations contribute to the pathogenesis of cerebral small vessel disease.Shared underlying pathological mechanisms, however, may also be a possible explanation for the associations we found between arterial stiffness and markers of cerebral small vessel disease.^2^

CVR was associated with cognition, as assessed by the MMSE and MoCA, in older adults, including those with normal BP, HTN-1, and HTN-2, and was lower in HTN-2 patients than in subjects with normal BP and HTN-1 patients. A reduced CVR is known to be correlated with microangiopathy severity, which increases vascular resistance.^33^ CVR is known to be influenced by atherosclerosis, and in this study, a reduction in CVR in response to hypercapnia was indicative of cerebral small-vessel disease,^34^^,^ 35 because subjects with cerebral artery stenosis were excluded from the present study.

The associations we found between arterial stiffness and lower breath-holding index were most pronounced in persons with more severe hypertension. Evidence suggests that in the presence of hypertension, structural and functional changes of the arterial wall occur earlier.^3^ Moreover, it is known that subjects with hypertension have a higher prevalence of cerebral small vessel disease.^36^^,^ 37

Our data suggest that within this group of subjects with severe hypertension, those with stiffer large arteries are more prone to having reduced cerebral vasoreactivity and lower cognitive performance.

On the other hand, the HTN-1 group had a higher rate of controlled BP and better cognitive performance in comparison to the HTN-2 group. Whether the antihypertensive treatment may have influenced the cognitive performance in this group is unclear.

So far, there is no convincing evidence from the trials identified that blood pressure lowering in late-life prevents the development of dementia or cognitive impairment in hypertensive patients with no apparent prior cerebrovascular disease.^38^ Most longitudinal studies have shown a significant inverse association between antihypertensive therapies and dementia incidence , particularly in Alzheimer Disease.^39^

Seven randomized, double blind placebo-controlled trials have evaluated the benefit of antihypertensive treatments on cognition. Three of these found positive results in terms of prevention of dementia (SYST-EUR)^40^ or cognitive decline (PROGRESS, HOPE).^41^^,^ 42 Others reported non-significant results (MRC, SHEP, SCOPE, HYVET-COG).^43^^-^ 46 This discrepancy emphasizes the difficulty in performing such trials: the follow-up has to be long enough to show a benefit, a large number of patients is needed for these studies, and for ethical reasons some antihypertensive treatments are often prescribed in the placebo group. Antihypertensive treatments could be beneficial for cognitive function by lowering blood pressure and/or by promoting specific neuroprotective effects. Three main antihypertensive subclasses have been associated with a beneficial effect on cognitive function beyond blood pressure reduction (calcium channel blockers, angiotensin converting enzyme inhibitor, angiotensin-AT1-receptor-blockers).^39^ Further long-term randomized trials, designed especially to assess a link between antihypertensive therapy and cognitive decline or dementia with cognition as the primary outcome are therefore needed.

In conclusion, severe hypertension was associated with higher arterial stiffness, worse breath-holding index, and lower cognitive performance. This scenario infers that hypertension severity is linked to higher arterial stiffness and lower vasoreactivity response, which may be associated with greater cognitive function decline in patients with chronic hypertension.

### Limitations.

First, our study sample was relatively small, and given the cross-sectional study design, association does not imply causation. Accordingly, our observations need confirmation in longitudinal and adequately powered studies. Second, our study was carried out in a select group of patients with hypertension referred to a university hospital, limiting the generalizability of our findings to other populations.

One limitation of the TCD method is related to the structure of the temporal window. The temporal window, where the TCD ultrasound probe is placed, thickens with age, making recording more difficult. A population-based study reported that 25% of the participants were lost because an adequate TCD signal could not be obtained, especially in older women.^35^^,^ 45 In the present study, 14.7% of participants were lost because of an inadequate TCD signal. These failure rates should be considered when planning TCD-based studies.
